# Playing Charades in the fMRI: Are Mirror and/or Mentalizing Areas Involved in Gestural Communication?

**DOI:** 10.1371/journal.pone.0006801

**Published:** 2009-08-27

**Authors:** Marleen B. Schippers, Valeria Gazzola, Rainer Goebel, Christian Keysers

**Affiliations:** 1 BCN NeuroImaging Center, University of Groningen, Groningen, The Netherlands; 2 Department of Neuroscience, University Medical Center Groningen, Groningen, The Netherlands; 3 Department of Cognitive Neuroscience, Maastricht University, Maastricht, The Netherlands; L'université Pierre et Marie Curie, France

## Abstract

Communication is an important aspect of human life, allowing us to powerfully coordinate our behaviour with that of others. Boiled down to its mere essentials, communication entails transferring a mental content from one brain to another. Spoken language obviously plays an important role in communication between human individuals. Manual gestures however often aid the semantic interpretation of the spoken message, and gestures may have played a central role in the earlier evolution of communication. Here we used the social game of charades to investigate the neural basis of gestural communication by having participants produce and interpret meaningful gestures while their brain activity was measured using functional magnetic resonance imaging. While participants decoded observed gestures, the putative mirror neuron system (pMNS: premotor, parietal and posterior mid-temporal cortex), associated with motor simulation, and the temporo-parietal junction (TPJ), associated with mentalizing and agency attribution, were significantly recruited. Of these areas only the pMNS was recruited during the production of gestures. This suggests that gestural communication relies on a combination of simulation and, during decoding, mentalizing/agency attribution brain areas. Comparing the decoding of gestures with a condition in which participants viewed the same gestures with an instruction not to interpret the gestures showed that although parts of the pMNS responded more strongly during active decoding, most of the pMNS and the TPJ did not show such significant task effects. This suggests that the mere observation of gestures recruits most of the system involved in voluntary interpretation.

## Introduction

Communication is an important aspect of human life, allowing us to powerfully coordinate our behaviour with that of others. Boiled down to its mere essentials, communication entails transferring a mental content from one brain to another. Spoken language obviously plays an important role in communication between human individuals. Manual gestures however often aid the semantic interpretation of the spoken message [1], [2], [3], [4], [5], and gestures may have played a central role in the earlier evolution of communication [6], [7], [8]. Therefore we will examine here the neural substrates of gestural communication in humans. Although this question has received less attention in the field of neuroscience than spoken language, two potentially complementary processes have been implicated in the perception and/or production of gestures: simulation and mentalizing [9], [10], [11].

The concept of simulation has received a surge of popularity since the discovery of mirror neurons in macaque monkeys [12], [13], [14], [15], [16], [17], [18], [19]. These neurons are active not only while the monkey performs an action (e.g. shelling a peanut), but also while the monkey sees or hears a similar action. Mirror neurons have been found in the ventral premotor and inferior parietal cortex of the monkey. However, it remains unclear whether other regions of the monkey brain contain mirror neurons for actions, because extensive single cell recording during both action execution and observation have so far not been performed outside of the premotor and inferior parietal lobule. Evidence for a similar system in humans has been derived from neuroimaging and transcranial magnetic stimulation studies [20], [21], [22], [23], [24], [25], [26], [27], [28], with the former showing that a network of areas is active both while people perform actions in the scanner and while they view or hear other people's actions. In humans, this system seems to include the dorsal premotor, somatosensory, cerebellar and posterior temporal cortex in addition to the ventral premotor, inferior frontal gyrus and inferior parietal lobule [21], [29]. These are the likely homologues of the aforementioned regions of the monkey [30], [31]. This extended set of areas can be called the putative Mirror Neuron System (pMNS) in order to emphasize that if a voxel in an fMRI experiment is involved in both execution and observation, the neurons within these voxels can, but do not have to, be mirror neurons [21], [32]: different populations of neurons within the same voxel could play the lead role during observation and execution. This caveat means that functional neuroimaging findings have to be interpreted with care: the fact that a region involved in action observation and execution is recruited during the processing of stimuli X might be suggestive of the fact that processing X involves ‘simulation’ (i.e. the recruitment of motor programs ‘as if’ the participant were producing these gestures him/herself) but it is not a guarantee that processing X truly depends on mirror neurons or simulation [33]. Neuroimaging therefore needs to ask questions in terms of brain regions (are regions of the pMNS involved?), and not in terms of cognitive processes involved (is simulation involved?): the former can be empirically measured using neuroimaging, the latter only tentatively suggested [34].

The discovery of mirror neurons has lead to the idea that we understand, at least in part, the goal-directed actions of others such as grasping and manipulating objects by activating our own motor and somatosensory representations of similar actions [15], [16], [19], [22], [35], [36], [37], [38], [39], [40], [41], [42], [43], [44], [45], [46] as if we had performed similar actions. This ‘as if’ component is why this process is called simulation. It seems that simulation occurs simultaneously at different levels of representations [11]: strictly and broadly congruent mirror neurons in the monkey for instance represent details of an action and the goal of an action, respectively and simultaneously [15], and experiments in human support the notion that both the details (TMS) and goals [32], [39] of actions are simulated. Whether the same system is involved in perceiving *communicative* gestures has been much less investigated.

Several lesion studies have investigated the neural basis of gesture production and perception in the context of apraxia. This is a disorder in which patients have difficulty with the control of action, including impairment in the production of gestures. In ideational apraxia, patients have preserved basic motor skills, but if asked to mimic the use of tools (e.g. show me how you would use a hammer to hammer a nail), they fail to produce the correct actions [47]. The ability to mimic is therefore traditionally used as a localizer for areas related to apraxia [48]. These studies have shown that the normal production of gestures requires an intact left posterior parietal lobe, including the parietal node of the pMNS [49], [50], [51], [52], [53], [54], [55], [56]. More recently, Montgomery, Isenberg, & Haxby [57] use a functional neuroimaging study to show that observing and producing *communicative* hand gestures activated the superior temporal sulcus, inferior parietal lobule and frontal operculum – a set of regions that corresponds to those of the pMNS. A limitation of this well controlled study is the fact that the participants had no genuine communicative intent: they produced pre-trained gestures in response to words (e.g. “thumbs up”) in the production condition, and passively observed stereotyped short movie clips of hand gestures in the observation condition. In addition, the authors intermixed imitation trials with passive observation trials. This may have lead to activations in motor production areas during gesture observation trials simply as a covert rehearsal of the motor programs that will later be needed for imitation. Overall, this task may therefore differ in important ways from the real life processes involved. For example, if one is in a foreign country, does not speak the language, and has only gestures to ask where to find a good restaurant. Would such a situation also primarily recruit the pMNS? Would other regions become important, including those involved in asking yourself what the other person is thinking, i.e. mentalizing areas?

A set of brain regions has been implicated in such reflection about the mental state of others. These areas include the medial prefrontal cortex (mPFC, in particular the paracingulate gyrus) and the temporo-parietal junction (TPJ) [58], [59], [60], [61], [62], [63], [64], [65], [66], [67], [68], [69], [70], [71]. Gallagher & Frith [72] compared the recognition of hand gestures expressing internal states (e.g. I feel cold) with those expressing a command (e.g. come here!). They additionally contrasted a *recognition* condition (was the gesture positive?) against an *observation* condition (which hand moved higher in the movie?). In particular, they report in the results and their Table 4 that the left anterior paracingulate cortex (putative BA32), thought to be a key node of the putative ‘theory of mind’ network (pToM area) appeared in an interaction contrast (recognizing expressive gestures – observing expressive gestures – recognizing orders+observing orders), and interpreted this finding as evidence for ToM involvement in interpreting gestures that express inner states. From the evidence presented in the report however, this interpretation is problematic, as they also report in the results and their Table 3, that the left anterior paracingulate cortex (putative BA32) is more active while *observing* gestures compared to *recognizing* them. While it is uncertain from the tables alone whether overlapping regions of the paracingulate cortex were present in these two contrasts, the paracingulate cortex was absent from the contrast recognizing – observing. This would be difficult to reconcile with the area being responsible for recognition. The involvement of ToM regions in gesture recognition therefore remains uncertain. In addition, although the TPJ is reliably recruited by tasks requiring mentalizing [61], [63], [68], [69], it is unlikely that this region specializes in attributing mental states to others: it is likely that it serves domain general functions relating to attention [73] and/or comparing sensory input with motor commands [74] which happen also to be important during mental state attribution.

The study described here explicitly investigates the role of both the pMNS and pToM areas by pioneering the use of a well-established gestural communication task into the field of neuroscience: the game of ‘charades’. We recorded brain activity while (romantically involved) couples played this game with each other. One partner would first be scanned while gesturing an action or object into a camera in the knowledge that his partner would later need to guess the action/object based on his recorded gestures. The other partner was to be scanned while decoding the gestures. The roles were then reversed. This allowed us to measure brain activity while people invent and execute gestures suitable to communicate a complex concept to another person, and while another person is decoding these gestures to guess the original concept. In addition, we examined if the brain activity recorded during this natural form of communication was specific for a communicative setting. We replayed the movies of their partner's gestures to each participant on a separate day, but this time, did not ask them to guess what their partner was trying to tell them. All participants reported finding the game very motivating, and experienced the experiment as a genuine and spontaneous form of communication.

Based on the idea that the pMNS might map the communicative actions of others onto the programs for producing similar actions, we hypothesized that parts of the areas involved in generating gestures would also become activated during the observation of communicative actions. To examine if this system overlaps with the pMNS for goal-directed actions, we examined if the pMNS as defined in previous experiments [39] becomes active both during gesture production and observation. Furthermore, several studies have shown the involvement of the TPJ and mPFC in tasks where people have to explicitly infer the mental states of another person. We therefore examined whether these pToM areas are involved during the charades game. Activity during gesture production may reflect a theory-of-mind of how the partner might interpret the gestures, and activity during gesture interpretation may reflect a theory-of-mind of what the partner might have meant while generating the gestures. pMNS and pToM areas could complement each other during the charades task [9], [10], [11]. The pMNS areas have been shown to be relatively stimulus driven independent of the task [e.g. 9], [75], while pToM areas seem more recruited during tasks that explicitly direct peoples minds to the mental states of others [9]. This line of reasoning would predict that pMNS areas would respond during the charades game and the control condition because they involved similar stimuli and motor actions. However, the pToM areas might respond during the charades game because this encourages mental state attribution but not during the control condition, which does not.

## Materials and Methods

### Participants

Twelve couples (total: 24 participants) were scanned while playing the game charades. The mean age of the participants was 27.5±3.8 years. Each couple consisted of a man and a woman involved in a romantic relationship for at least 6 months. As in previous studies on emotional empathy [76], we included this criterion not to study romantic relations specifically but to maximise the social relevance of this experiment because we expected couples to be more motivated, more at ease, and to have a better or faster understanding of each other's gestures than a strangers do. Participants were asked to fill out a questionnaire about their neurological and medical history including whether they had metal objects in their body. This is a standard procedure to ensure the safety of the participants whilst in the scanner. Participants were also asked not to drink coffee before scanning commenced. The participants freely consented to participating in the study by signing an informed consent form and were scaled for their right-handedness on the Edinburgh Righthandedness scale [77]. This entire study was approved by the Medical Ethics Committee of the University Medical Center Groningen (2007/080).

### Task/Experimental Design

The experiment consisted of two separate sessions on different days. In the first session, the couple was required to play the game of charades. In the second, detailed anatomical scans and a control condition were acquired. For the game of charades, participants took turns going into the scanner, alternating gesturing and guessing of words. Words were either objects (for example nutcracker, watch, pencil sharpener) or actions (for example painting, knitting, shaving, see Tab. 1). Each participant performed two gesture and two guess runs in which they gestured and guessed 14 words in total (7 per run). The set of words used was the same for each couple, but word order was randomized between participants. After the last gesture-session, a T1 image was acquired.

**Table 1:** Action and object words used in the game.

Actions		Objects	
peel fruit	fold	nutcracker	telephone
ride a bike	drive a car	pencil sharpener	winding stairs
shuffle cards	play the piano	pistol	ashtray
polish nails	squeeze fruit	electric eel	bow
juggle	paint	watch	handcuffs
knit	light fireworks	board game	glove
throw a snowball	shave	canoe	cork screw

#### Gesture run

During a gesture run, the participant was presented with a word on the screen and was instructed to communicate this word to his or her partner by means of gestures. Every word had to be gestured for 90 seconds. Prior to scanning participants were trained not to repeat the same gesture over and over again, but to keep generating new gestures to provide their partner with multiple sources of information. The participant could see how much time he/she needed to keep gesturing by a progress bar on the screen. A fixation cross was presented for 20 s after each word, which served as our baseline. The gestures were recorded from the control room of the MR-scanner with a video camera (Sony DSR-PDX10P). After the participant had gestured seven words, he/she was taken out of the scanner and went into the waiting room, while his/her partner went into the scanner to guess what he/she had gestured. During this changeover, the experimenter cut the recording of the gestures into movies of 90 s in which the participant gestured a word (see supplementary information for an example of a gesture recording, movie S1). To ensure that the movies were cut at exactly the moment the word was presented to the gesturing participant, the stimulus computer's sound card emitted a sound at the beginning of word presentation. The output of the sound card was connected to the audio input of the video camera, thus allowing the auditory signal to serve as a marker for cutting. To minimize the amount of head motion in the participants, the upper arms of the participant were fixed to the bed by means of a Velcro strap band. This left the participants free to gesture with their lower arms, hand, and fingers, which was sufficient to ensure 86% percent correct gesture recognition.

#### Guess run

During a guess run, the participant was shown the movies that were recorded in the gesture run of their partner. The task they had to perform was to guess what their partner was trying to gesture to them. Participants were asked to consider the gestures for at least 50 seconds before committing to a specific interpretation of the gestures. This was done to ensure at least 50 seconds of data in each trial to examine the time course of activity (i.e. is brain activity in region X sustained for as long as participants are interpreting the gestures?). This was done by showing a progress bar under the movie, changing from red to green after 50 seconds, indicating the beginning of the period (50–90 s post stimulus onset) during which participants could decide on their interpretation of the gestures, whenever they felt confident. After the button press with which the participants indicated to be ready to respond, a multiple choice menu was presented. In this menu they had to choose the correct word from five alternatives. One of the alternatives was always ‘none of the above’ and the correct answer was always present in the multiple-choice menu. The correct answer was never the option ‘none of the above’. This marked the end of a trial. Two consecutive trials were separated by 20 seconds of a white fixation cross against a black background, which served as our baseline.

#### Passive observation run

As a control condition for the guess run, the participants watched the movies again which they had seen during the guessing condition. This time, they were instructed not to guess what was gestured, but only to passively view them. To keep the run exactly the same as the original guess run, the movie would stop at the moment the participant during the original run had pushed the button. The same multiple-choice menu would appear and the participant had to answer again. This time, however, they had to select the word written in green letters. The green word was the correct answer. A fixation cross was presented between two consecutive trials for 20 seconds and served as our baseline.

### Data Acquisition

Functional imaging data was recorded with a Philips 3.0 T MR scanner, using gradient echo planar imaging (EPI). T2* weighted images revealed changes in blood oxygen level. Repetition time was 1.33 seconds. The whole brain was scanned in 28 (axial) slices with a thickness of 4.5 mm. In the first session, a fast structural image (“fast anatomy”) was acquired of the participant's brain, while in the second session an additional structural image of higher resolution was acquired. Both were structural, T1-weighted images.

### Data Analysis/Statistical Analysis

**Figure 1:**
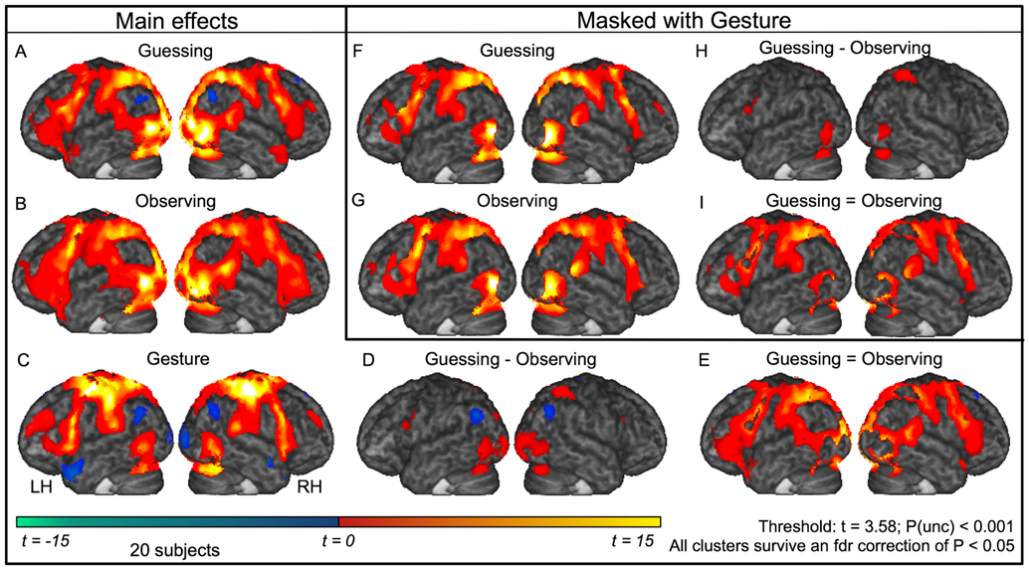
Activation maps rendered on mean anatomy. Activation maps rendered on the mean anatomy of all 20 subjects. (A–D) Main effects guessing-baseline, passive observation-baseline, gesture-baseline, guessing-passive observation. (E) Areas similarly activated during guessing and passive observation (i.e. guessing-baseline p<0.001 & passive observation-baseline p<0.001 & guessing-passive observation p>0.001). (F–I) A, B, D and E, each masked inclusively with C. All images are thresholded at t = 3.58 which corresponds to an uncorrected p≤0.001. All voxels also survive false discovery rate (fdr) correction p<0.05.

Data were analyzed using the Statistical Parametric Mapping Software, version 2 (SPM2). EPI data were corrected for slice timing and realigned. The T1 image was co-registered to the mean EPI and segmented, the normalization parameters to normalize the gray-matter segment onto the MNI gray-matter template were determined, and applied to all the EPI images. Normalized EPI images were then smoothed with a Gaussian kernel of 10 mm. Three general linear models were estimated: one for the gesturing, one for the guessing and one for the passive observation sessions. All words, whether they were actions or objects, guessed correctly or incorrectly, were modelled together in one condition. The predictor in the gesture run consisted of the whole period during which the gesture was executed (90 s). In the active guessing and passive observation runs two predictors were included in the general linear model: (a) the period in which the movie was shown until button press and (b) from button-press until the participant had given the answer. All predictors were convolved with the hemodynamic response function. Each participant's mean parameter estimates were then tested at the second level (one-sampled t-test). Activations are displayed on a mean anatomical image of all participants (see Fig. 1). To examine differences between object words and action words, the data was also modelled using separate predictors for the two categories but the contrasts ‘guessing objects–guessing actions’, and the reverse contrast, were not significant at p<0.05 (FDR corrected) in any voxel. Therefore only analyses using a single predictor are reported here. The same applies to the gesture analyses. To control for head motion, we included six motion parameters as covariates of no interest (translation and rotation in x, y and z directions) and excluded four participants, who moved more than the voxel size (3.5×3.5×4.5 mm). Thus, the analyses and results presented in this paper are based on 20 participants.

### Comparisons Guessing vs Passive Observation

Given that passive observation always had to be acquired after guessing, differences between these conditions could in theory be linked, amongst others, to systematic differences in the MR-signal across sessions. We examined this possibility by calculating average global maps for each participant (i.e. a contrast with ones in the last columns of the SPM design matrix for the two sessions). These maps were compared in a paired t-test. There were no significant differences at p<0.05 (FDR corrected).

### Localizing shared circuits

We define shared circuits as those voxels that are active both during an execution *and* an observation condition. This was done by thresholding the group-level analysis of the gesturing condition (vs. passive baseline) at p<0.001 (uncorrected) to create a binary map (all above-threshold voxels have the value 1 and all the other have the value 0) and applying this image as a mask in the second level analysis of guessing or passive observation.

### Putative Mirror Neuron System ROIs

**Figure 2:**
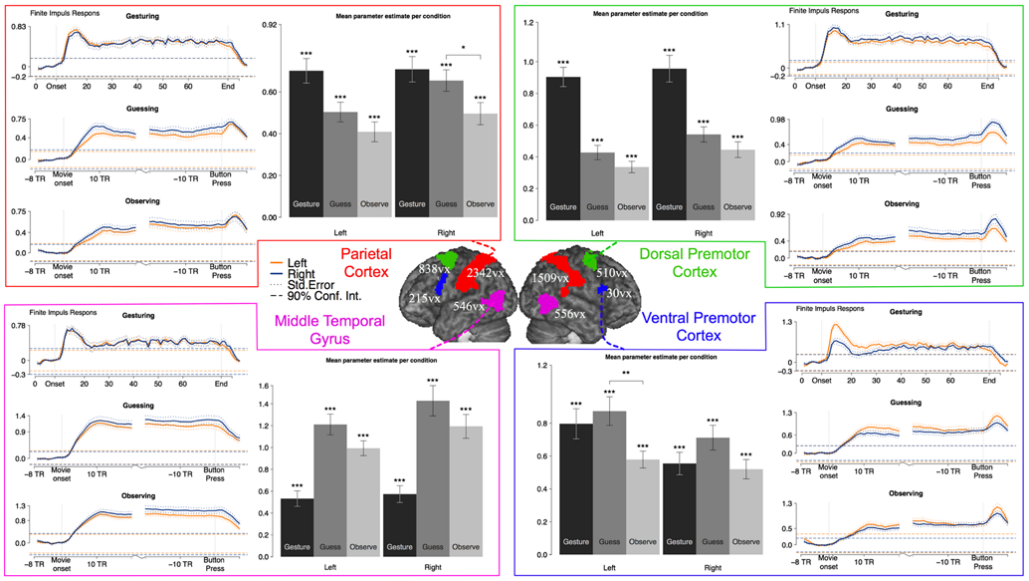
ROI analysis results for the pMNS areas. Locations and sizes of the pMNS ROI (center) together with their parameter estimates for each condition (bar graphs). Curves show the peri-stimulus time histogram for each condition in each ROI. For gesturing, the whole period of gesturing is plotted, from 8 volumes before the onset of the gesture until 8 volumes after the gesture has stopped. During both guessing and passive observation, the begin period (8 volumes before onset of the movie of the gesture until 20 volumes after) and the end period (20 volumes before button press until 8 volumes after) are plotted in the same graph, with the interruption due to the participants responding after variable amounts of time. See centre legend for further details.

The areas which together form the mirror neuron system were defined based on a previous study done in our lab with 16 participants [39]. In this study, healthy participants observed and performed goal-directed hand actions. The subset of areas that are active both during the execution and the observation condition form the pMNS. The areas included a section of the ventral-and dorsal premotor cortex, the parietal lobe (including Brodmann Area (BA) 2 and the cortex along the intraparietal sulcus and the supramarginal gyrus) and the middle temporal gyrus (see Fig. 2 for location and size of the rois).

### Putative Theory of Mind areas ROIs

The medial prefrontal cortex and the temporo-parietal junction are considered typical theory-of-mind areas. We included both these areas in our analyses. We based the ROIs in the medial prefrontal cortex on the review article of Amodio *&* Frith [78] in which different tasks are outlined that lead to activation in this area. Based on this meta-analysis, we drew our ROI in the anterior rostral medial frontal cortex. Activations in this region are associated with mentalizing, person-perception and self-knowledge. This roughly corresponds to Brodmann area 10. We used the Talairach coordinates from that article to hand-draw a quadrilateral ROI (from (−2,34,5) and (−2,26,15) to (−2,71,5) and (−2,55,44) respectively). This triangular shape started medially (at X = ±2) and extended laterally 13 mm to cover the grey matter (until X = ±15). To fit the ROI in the best possible way to our participants' data, we multiplied this hand drawn image with a thresheld mask (>0.3) of the mean grey matter segment that was obtained through segmenting the brain of each individual participant.

**Figure 3:**
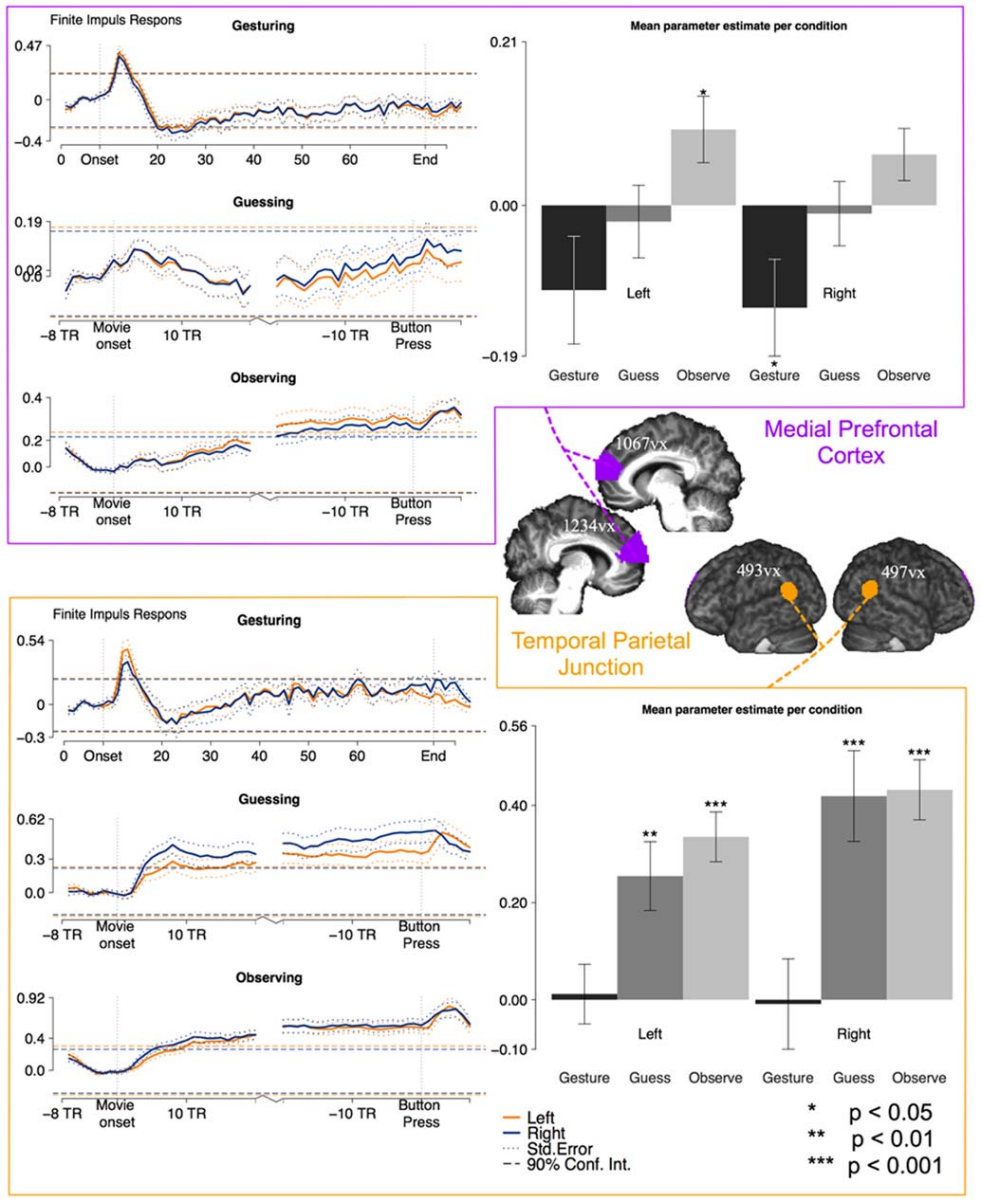
ROI analysis results for the pToM areas. Same as Fig. 2 for pToM areas.

In a similar fashion we defined the temporal parietal junction on the basis of coordinates mentioned in Mitchell [73]. Mitchell [73] gives an overview of all different peak coordinates associated with the temporal parietal junction. To construct our ROI, we calculated the mean of these three coordinate-pairs ((54,−51,18), (54,−54,24), (60,−57,15)) and used this as the centre point of a sphere with a radius of 10 mm sphere. Again, we multiplied this with the mean grey matter segment to exclude out-of-brain voxels as much as possible. For the location and sizes of these regions of interest, see Figure 3.

### Calculating the finite impulse response for the ROIs

For each ROI, we extracted the average BOLD response around two events of interest: the onset of a gesture and the moment the button was pushed when the word was guessed. During guessing and passive observation 28 peri-stimulus timebins were extracted, in which each bin had the same length as the repetition time (1.33 s). The signal was extracted from the period commencing 8 bins before gesture onset and continuing until 20 bins following it. The same was done for the button press, including 20 bins before and 8 bins after. During gesturing, the average BOLD response was extracted for the whole period in which the gesture was performed, starting at 8 bins before the onset and lasting for 84 bins. The MarsBar toolbox in SPM2 was used for this extraction [79]. This modeling resulted in para-stimulus time histograms, which show the development of brain activity over time (see Fig. 2–3).

### Thresholding

All final whole brain analysis results are thresheld at p<0.001 (uncorrected). Only clusters that additionally survived a false discovery rate correction at p<0.05 are reported. This means that all whole brain results presented in this manuscript survive fdr correction at p<0.05, but are presented at p<0.001 (uncorrected) because this turned out to be the most stringent of the two. Note that in the case of masking, the correction is only applied after the masking. Given that the mPFC failed to show significant activation at these thresholds, we additionally performed a small volume corrected analysis at p<0.05 within the volume defined as our mPFC ROI to challenge our negative findings.

For the regions of interest analysis, we specify the significance of any difference with p<0.05. This was done for the reader to have the freedom to challenge negative findings at a permissive threshold (p<0.05), while at the same time providing more stringent evidence for the key positive results.

## Results

### Behavioural Results

During guessing the participants were asked to consider each movie for at least 50 seconds after which they could push the button when they thought they knew what was being gestured to enter the multiple-choice menu. The average latency to response was 58 seconds. Participants were equally accurate on both categories: 88% of the object words were guessed correctly against 85% of the action words (t (41) = −0.79, p>.43). We did not find a significant difference between the two types of gestures, neither in terms of latency to respond (58 s±11 s for action and 59 s±12 s for object words, t(330) = −1.33, p>.18) nor in terms of accuracy (6.13±0.74 sd correct out of 7 action and 5.92±1.05 sd correct our of 7 object words, t(41) = −0.79, p>.43). Words that were guessed incorrectly were watched significantly longer than words that were guessed correctly: 58 s±11 s for the 289 correct guesses versus 65 s±14 s for the 47 incorrect guesses (t (56) = −3.48, p<.001).

### Whole Brain fMRI Results

#### Main effects of guessing

**Figure 4:**
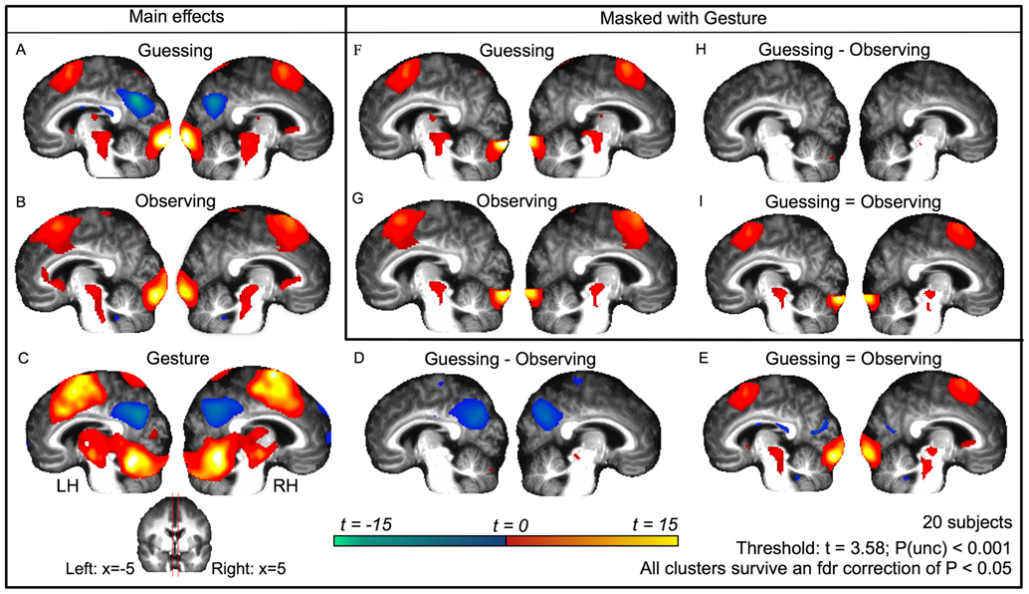
Activation maps rendered on medial wall of mean anatomy. Same as Fig. 1, but activations are now shown on the left (x = −5) and right (x = 5) medial wall of the mean anatomy of the 20 subjects.

**A**ctivation clusters during guessing compared to baseline are shown in Table S1 and Figure 1A. Of particular interest were the clusters of activity found along the precentral gyrus (BA 6) and extending into the inferior frontal gyrus (BA 44 and 45), in the middle and superior temporal areas (including the TPJ), the primary somatosensory cortex (BA 2 in particular) and the supramarginal gyri. Inspection of the medial wall (see Figure 4) revealed activations in the superior medial gyrus in what Amodio and Frith [78] call the posterior section of the rostral medial frontal cortex but not in the anterior section associated with theory-of-mind (our mPFC ROI). During this condition, reductions in the BOLD signal were found in the precuneus, right insula, and bilaterally the angular gyrus and the operculum (OP 1 to 4). There were no differences in activation when object words are compared with action words or vice versa (not shown).

#### Main effects of passive observation

Table S2 and Figure 1B show activation clusters during passive observation compared to passive baseline. Clusters of activity were found in locations very similar to those during active guessing, including BA 6, 44, 45, 2, middle and superior temporal areas (including the TPJ), and supramarginal gyri. Inspection of the medial wall (see Figure 4) revealed activations in the superior medial gyrus and adjacent middle cingulate gyrus in what Amodio and Frith [78] call the *posterior* section of the rostral medial frontal cortex but not in the *anterior* section associated with theory-of-mind (our mPFC ROI). Reductions in the BOLD signal were found in the precuneus, the caudate nucleus and two small clusters in the cerebellum.

#### Main effects of gesturing

**A**ll activation clusters during gesturing compared to a passive baseline are shown in Table S3 and Figure 1C. Notably, clusters of activity were found in the primary, pre- and supplementary motor areas (BA 4a/p and 6), BA 44 and 45. Both inferior and superior parietal lobules were involved, together with somatosensory cortices and the middle and superior temporal gyri (including the TPJ). Inspection of the medial wall (see Figure 4) revealed activations in the superior medial gyrus and adjacent middle cingulate cortex in what Amodio and Frith [78] call the posterior section of the rostral medial frontal cortex but not in the anterior section associated with theory-of-mind (our mPFC ROI). Instead, the most anterior sections show evidence of reduced BOLD relative to baseline. Extensive clusters were found in the precuneus, the angular gyrus bilateral, the medial prefrontal cortex and the left temporal pole, which were more active during the baseline than during gesturing. Additional reductions in BOLD signal were found in the more posterior superior parts of BA 17 and 18 and in the right hippocampus and amygdala.

#### Similarities and differences between guessing and passive observation

The comparison of activity between guessing and passive observation is rendered more difficult by the fact that they were acquired in separate sessions, and results should be considered with care. Counterbalancing the order of acquisition would however have interfered with the aims of the experiments for two reasons. First, an instruction not to engage in active guessing would be even more difficult during a passive observation trial if participants would know that they later need to guess the meaning of the same movie. Second, capturing the neural processes involved in interpreting gestures in an ecologically plausible way would be disturbed by ‘passively’ viewing the movies before. Using different movies for passive observation and active guessing would not be a solution either because the stimuli might differ in important ways.

To exclude the possibility that differences in brain activity between guessing and passive observation could simply be due to systematic differences in the state of the scanner, we additionally compared the mean fMRI signal between the two sessions (using a two-sample t-test comparing the globals in the two sessions, see Methods). No region in the brain showed such an effect under a threshold of p<0.05 (FDR corrected). This means that functional differences cannot be due to differences in the mean signal alone.

Two analyses were then performed to compare brain activity during the processing of the same movies during active guessing versus passive observation: one to map differences and one to map similarities between the two conditions. Areas, which were recruited to a greater extent during guessing than during passive observation were as follows: the inferior and middle temporal gyri and areas V5/MT+bilaterally, and more anterior in the brain a cluster in BA 44. Again, inspection of the medial wall (see Figure 4) showed no clusters of activation in the mPFC ROI associated with theory-of-mind. Differences due to a greater involvement during passive observation than during guessing were located in the angular gyrus and the precuneus. These were areas that were deactivated compared to the passive baseline in the main effects. A full description and visualization of the areas can be found in Table S4 and Figure 1D. In contrast, much larger areas were recruited during both active guessing and passive observation without significant difference between these conditions. These included the precentral gyrus (BA 6) and BA 44 and 45, the somatosensory cortex (BA2), the inferior parietal lobule, and the middle and superior temporal areas. For a full description and visualization of the areas, see Table S5 and Figure 1E.

#### Guessing masked with gesturing, passive observation masked with gesturing (shared circuits)

We defined shared circuits as voxels recruited both during the execution and the observation of gestures. Masking the activity during guessing with the activity during gesturing shows, among others, shared recruitment of the following areas: the precentral gyrus (BA 6) extending into the inferior frontal gyrus (BA 44 and 45), the primary somatosensory cortex (BA2 in particular), the middle and superior temporal areas and the supramarginal gyri. Roughly the same pattern emerges when the activity during observing is masked with the activity during gesturing. Figures 1F and 1G detail these activations.

#### Similarities and differences between guessing and passive observation masked with gesturing

**C**ontrasting active guessing with passive observation and masking this with the activation during gesturing shows noticeable peaks in the right inferior parietal lobule and in the left BA 44 (Fig. 1H). Substantially larger areas remain when the activity that is present during both active guessing and passive observation is masked with activity during gesturing, without there being a significant difference between these conditions. These include much of the somatosensory, premotor, middle temporal- and supramarginal cortex (Fig. 1I).

### Regions of Interest fMRI Results

#### Putative mirror neuron system (Figure 2)

The bar plot of the parameter estimates during the different conditions show that all conditions activate all putative mirror neuron areas significantly even at an uncorrected threshold of P<0.001. The time courses show further that all areas are substantially activated during the whole period of each condition (as evidenced by the mean activity exceeding the confidence interval (dashed line) of the mean activity during the 5 volumes prior to stimulus onset). Two of these areas make a significant distinction between guessing and passive observation, but only under an uncorrected threshold of P<0.05. These areas are the right parietal cortex and the left ventral premotor cortex.

#### Putative theory-of-mind areas (Figure 3)

The medial prefrontal cortex shows no significant response to any of the conditions when applying an uncorrected threshold of P<0.001, in contrast to the temporo-parietal junction. The time courses confirm this observation: activation almost never reaches significantly above the baseline activity, except at the end of a movie during the passive observation condition. The temporo-parietal junction is recruited significantly during both guessing and passive observation, but not during gesturing. This is also confirmed in its time courses.

## Discussion

In this experiment romantically involved couples played the game of charades in the scanner, taking turns as either the sender (gesturing) or receiver (guessing) of gestures. In this motivating context, they very naturally generated and decoded novel gestures with a communicative intention. The main goals of the study were to investigate to what extent (a) the pMNS for transitive hand actions and (b) pToM areas are involved in deliberate communication through gestures, and (c) how dependent the activity in these areas is on the communicative intention induced by the task. We analyzed the involvement of these two networks in two ways: through a whole-brain and a region-of-interest (ROI) analysis. Both analyses gave similar results. The pMNS does indeed become activated during communication through gestures, with highly overlapping brain areas involved in sending and receiving the gestural message. In contrast, the most typical of pToM areas, the anterior rostral medial frontal cortex associated with theory-of-mind [78] (which we will refer to as mPFC) was not recruited beyond baseline levels during either sending or receiving gestural messages; the TPJ was engaged during observation but not during gesturing. The pMNS and TPJ were significantly activated both during guessing and passive viewing. The hypothesis that the TPJ would only be activated during the guessing conditions that explicitly encourages decoding the mental states (i.e. what is he trying to tell me?) but not the control condition (passive viewing), was not confirmed.

### Involvement of the putative mirror neuron system

Our study shows that brain regions associated with the pMNS for goal-directed, transitive actions were recruited during gestural communication - even when physical objects are not being present. A whole-brain analysis, in which the execution of gestures is used to mask the guessing or passive observation of gestures, shows a large overlap between the areas recruited in the three conditions (Fig. 1F,G). Furthermore, the ROI analysis of the pMNS, as defined using actions directed at objects [39], shows sustained activity in these areas during the whole period of gesturing, guessing and passive observation (Fig. 2). Combining the study of Gazzola, Rizzolatti et al. [39] with the results of the current study show that the same set of voxels in the brain is therefore involved in (a) mapping the object-directed hand actions of others onto the neural substrates involved in executing similar object-directed hand actions and (b) mapping the gestures of others onto the neural substrates involved in executing similar gestures. This extends previous findings [57] by showing that even in the absence of imitation trials, and during a genuinely communicative task, the brain regions associated with the pMNS for goal-directed actions are consistently activated. See online Supporting Information (Text S1) for a discussion of how this finding relates to the question of whether the pMNS requires objects to be activated.

To maintain the flow of the game, control conditions involving the static vision of hands or meaningless hand actions were not included in this study. One might therefore question whether the activity found in the ROIs during gesture viewing (guessing or passive observation) is specific to actions or whether it reflects unspecific attentional resources. The ROIs used to extract the signal in the pMNS have been extensively examined in our laboratory using the same scanner and analysis software [21], [32], [39]. Figure S1 (see online Supporting Information) illustrates the peak percent signal changes of the time courses measured in Gazzola, Rizzolatti et al. [39, their Fig. S3] and those observed during the same time period of the gesture condition in the present experiment. Doing so revealed that activations in the guessing condition here exceeded those of the control conditions of Gazzola, Rizzolatti et al., [39] in all but the right ventral premotor ROI. Indeed, in the same ROIs, the activity in the present experiment often exceeded even the vision of goal directed actions in all but the right ventral premotor ROI. Although comparisons across experiments are problematic and should be interpreted with caution, this does suggest that the activity during the viewing of gestures in the present experiment reflects genuine action processing that exceeds that during the sight of mere movements.

Interestingly, the brain activity induced while engaged in active guessing overlapped considerably with that obtained during the second showing of the exact same visual stimuli but without the task (Fig. 1F,G). As noted in the results, quantitative comparisons across different sessions are problematic, and conclusions drawn from these comparisons have to be considered with care. A quantitative comparison between activity in the two conditions within the confines of regions involved in gesture production however did reveal significantly higher BOLD during active guessing compared to passive viewing. The areas particularly involved were BA44 and the MTG (Fig. 1H). These differences are unlikely to be due to systematic differences in the sensitivity of the scanner, as there were no significant differences in these areas between the globals extracted by the general linear model on the two scanning days (see methods). These differences were also marginal compared to the much more extensive network of premotor, parietal and temporal regions of the pMNS that did not show a significant difference between the two tasks (Fig. 1I). This finding is in line with a previous study which showed that the pMNS for facial movements is only marginally affected by task [75]. A number of studies [80], [81] have shown that observing other people's behaviour interferes with the observer's own movements even if it would be beneficial for the observer to ignore the movements of the other person. We believe that the similarity between the activity in passive viewing and active guessing, and the fact that both significantly activate the pMNS, highlights the tendency of the pMNS and/or the subjects to process the actions of others even if the experimenter's instructions do not explicitly encourage them to do so. With ‘and/or the subject’ we refer to the fact that upon debriefing, some of our participants reported finding it hard to refrain entirely from interpreting the gestures in the passive viewing condition. They did report however, that they interpreted the actions more during the guessing condition.

It should be noted that activation of the pMNS regions during gesture observation and production can, but does not have to reflect activity in mirror neurons within these voxels. This is because a voxel involved in two tasks could contain a population of neurons involved in both, as has been shown in the monkey [15], [16], [17] and/or two distinct populations, each of which being involved in only one of the two tasks, interdigitated within the volume of the voxel [21].

### Involvement of Theory-of-Mind areas

Because playing charades could require the explicit guessing of the communicative mental state of the gesturer (“what was he trying to tell me?”), our second experimental question was whether pToM areas, including the mPFC and the TPJ, would be significantly recruited during the gesturing, active guessing and/or passive viewing.

#### Medial Prefrontal Cortex

Previous studies have shown that mentalizing is associated with activity in the mPFC [58], [59], [63], [64], [65], [71], [82], [83], [84]. More specifically, Sommer et al. [69] showed that true belief reasoning (which might be closer to what participants need to do here compared to false-belief reasoning) involves the mPFC. Furthermore, Kampe, Frith, & Frith [85], as well as Walter et al. [71], and Ciaramidaro et al. [60] found the anterior paracingulate cortex to be recruited while recognizing the communicative intentions of others [for reviews see 62,78]. In our experiment, neither the ROI nor the whole brain analysis revealed activations above baseline in the mPFC during any of the conditions. This was true using a threshold of p<0.001, and for the ROI analysis at using p<0.01 (see Fig. 3). This negative finding suggests that the mPFC may not play an active role in gestural communication. This finding seems different from Gallagher & Frith's [72] conclusions that the left anterior paracingulate cortex was selectively more involved in recognizing gestures expressing inner states. This difference may be due to the fact that our gestures referred to objects (nutcracker) and object-directed actions (riding a bicycle) while Gallagher & Frith's expressive gestures referred to inner states (I feel cold). Thinking about the inner states of others is indeed known to be particularly effective at triggering mPFC activity [78].

We asked participants to consider the movies of their partner's actions for at least 50 seconds before reporting their interpretation of the gestures. This requirement was established to ascertain sufficient data points to examine the time course of activity. A consequence of this requirement, however, is the participants may have guessed the meaning of the gestures early in the trial, and before they gave their answer. Could the lack of mPFC activity in the whole-brain and ROI analysis be due to these trials? We believe not. If this were the case, the time course extracted from the mPFC ROI during the guessing condition should exceed the baseline activity or that during observation condition at least early in the trial. Our data (Fig. 3) does not support this hypothesis.

It should be note however, that all conditions in our experiment were compared against a passive baseline. It has been argued that a seemingly passive baseline actually goes hand-in-hand with increased metabolism in the mPFC [86], possibly because of self referential processing. Such default, self-referential activity would have been suspended by our tasks, leading to a decrease in mPFC activity that may have masked mentalizing processes of comparatively smaller metabolic demands.

#### Temporal Parietal Junction

We found that the TPJ was significantly activated during guessing and passive observation but not gesturing. The TPJ has been associated with the ability to mentalize [68], [87], [88], [89], but other studies suggest that this involvement might reflect attentional reorientation necessary for mentalizing rather than mentalizing per se [73], [74]. It therefore remains unclear what can be deduced from its activation in some of our conditions. It might be that activity truly reflects mentalizing [90], suggesting that the decoding of gestures but not their generation requires mentalizing. What sheds doubt on this interpretation is that during mentalizing tasks, the TPJ typically coactivates with mPFC, and this coactivation may be more unique for mentalizing than the activity of either region taken alone. Alternatively, activity in the TPJ may reflect attentional reorienting [73], [74] (for instance between the gestures as an outer stimulus and the hypothesis about their meaning as an inner stimulus), which gesture interpretation may share with mentalizing. Finally, some have interpreted TPJ activity during the attribution of agency [74], an interpretation that would match our finding TPJ activity only during to the third person conditions (guessing and passive observation) Further experiments are needed to disentangle these alternatives.

## 

### Conclusions

The putative mirror neuron system (pMNS) is recruited by observing communicative gestures (both with and without an instruction to interpret) and by the production of similar gestures. In contrast, the mPFC, which is often associated with mentalizing and ToM, was not recruited above baseline during gestural communication. Finally the TPJ, which is associated with mentalizing but also attention reorienting and the attribution of agency, was recruited during both passive observation and guessing. This suggests that observing gestures recruits a combination of TPJ and pMNS both when participants actively decode gestures and when they passively watch them. The pMNS - but not the TPJ - is recruited during the generation of similar gestures. These findings are in accordance with the idea that gestural communication could build upon a pMNS for goal-directed hand actions [6], [7]. The pMNS could create a simulated first person perspective of the gestures through a combination of forward and reverse models in the somatosensory and motor domain [21]. This simulation could then provide additional information for associating the vision of gestures to their meaning. Evidence for mentalizing during gestural communication in this experiment is weak however. During gesture interpretation, TPJ activity could reflect the fact that information from the pMNS could feed into pToM components (the TPJ) [9], [10], [11], but it is unclear why the mPFC would not have been active if activity truly reflects mentalizing. During gesture generation, neither the TPJ nor the mPFC were active above baseline. Alternatively, TPJ activity during gestural interpretation may reflect the attribution of agency to the action representations in the pMNS [74].

We have introduced the game of charades in neuroimaging research as a motivating social game to study gestural communication. This provides a new tool to study the involvement of pMNS in a genuinely communicational context. By extending this method to study virtual or neurological lesions it can be determined whether these regions play a *necessary* role in understanding and generating communicative gestures. A number of studies using gesturing tasks have found impairments in gesture recognition following motor skill impairment [91], [92], [93]. This suggests that the pMNS may indeed play a critical role. A recent study [92] shows that premotor and parietal lesions that impair *hand* action execution (as compared to mouth action execution) selectively impair the recognition of hand gestures (and their sounds). This confirms that lesions in the pMNS can selectively affect the production and perception of *particular* motor programs. This finding would be expected if simulation were important in gestural communication given that the pMNS is roughly somatotopically organized [22], [38], [94], [95]. Nevertheless, although gesture recognition is impaired in apraxic patients, performance typically remains substantially above chance level, suggesting that the pMNS cannot be the only route to associate gestures with meaning. Understanding the complementary nature of various sources of information within the brain during gestural communication will be an important focus of future research [9], [10], [11].

## Supporting Information

Text S1 Does the MNS need objects to be activated? Some studies have investigated whether the MNS can respond to actions not directed at objects. In this supporting information we discuss the question whether the current study can provide further insights into this question.
(0.04 MB DOC)

Figure S1 Comparison with Gazzola et al., 2007. Comparison of mean percent signal change during gesture observation (light and dark blue bars) with those during the observation of goal directed actions (red and orange, Gazzola et al., 2007). The blocks of action observation differed across experiments: over 50 s in the current experiment and 13.5 seconds in Gazzola et al., 2007. Instead of comparing parameter estimates over the entire period of observation, we therefore extracted the mean percent signal change at the moment (16 s) in which activity to the shorter of the two blocks (Gazzola et al., 2007) peaked. The bar graphs represent the mean percent signal change at 16 s post stimulus onset (±s.e.m.) separately for Guessing (dark blue) and Passive Observation (light blue) from the current study and for the observation of a hand manipulating an object (red) and a hand moving to rest on a table without manipulating an object (orange) from the data of Gazzola et al., 2007. ROIs are shown in the centre.
(7.51 MB TIF)

Table S1 Activation table for Guessing - Baseline. Table specifying for each supra-threshold cluster of activation during the contrast Guessing-Baseline, the t-value, location, anatomical description and, when available, probabilistically determined Brodmann area according to the anatomy toolbox (Eickhoff et al., 2005).
(0.02 MB XLS)

Table S2 Activation table for Passive Observation - Baseline - Table specifying for each supra-threshold cluster of activation during the contrast Passive Observation-Baseline, the t-value, location, anatomical description and, when available, probabilistically determined Brodmann area according to the anatomy toolbox (Eickhoff et al., 2005).
(0.02 MB XLS)

Table S3 Activation table for Gesturing - Baseline. Table specifying for each supra-threshold cluster of activation during the contrast Gesturing-Baseline, the t-value, location, anatomical description and, when available, probabilistically determined Brodmann area according to the anatomy toolbox (Eickhoff et al., 2005).
(0.01 MB XLS)

Table S4 Activation table for Guessing - Passive Observation. Table specifying for each supra-threshold cluster of activation during the contrast Guessing-Passive Observation, the t-value, location, anatomical description and, when available, probabilistically determined Brodmann area according to the anatomy toolbox (Eickhoff et al., 2005).
(0.01 MB XLS)

Table S5 Activation table for Guessing equals Passive Observation. Table specifying for each supra-threshold cluster of activation during the contrast Guessing equals Passive Observation, the t-value, location, anatomical description and, when available, probabilistically determined Brodmann area according to the anatomy toolbox (Eickhoff et al., 2005).
(0.02 MB XLS)

Movie S1 Example of a gesture recording. Example of the recorded gesture ‘boardgame’.
(7.73 MB MOV)

## References

[1] Iverson JM, Goldin-Meadow S (1998). Why people gesture when they speak.. Nature.

[2] Kendon A (1994). Do Gestures Communicate? A Review.. Research on Language and Social Interaction.

[3] McNeill D (1992). Hand and Mind: What Gestures Reveal about Thought.. University Of Chicago Press.

[4] Melinger A, Levelt WJM (2004). Gesture and the communicative intention of the speaker.. Gesture.

[5] Willems RM, Ozyürek A, Hagoort P (2007). When language meets action: the neural integration of gesture and speech.. Cerebral Cortex.

[6] Gentilucci M, Corballis MC (2006). From manual gesture to speech: a gradual transition.. Neuroscience and Biobehavioral Reviews.

[7] Rizzolatti G, Arbib MA (1998). Language within our grasp.. Trends in Neurosciences.

[8] Arbib MA (2008). From grasp to language: embodied concepts and the challenge of abstraction.. Journal of Physiology - Paris.

[9] de Lange FP, Spronk M, Willems RM, Toni I, Bekkering H (2008). Complementary systems for understanding action intentions.. Current biology.

[10] Keysers C, Gazzola V (2007). Integrating simulation and theory of mind: from self to social cognition.. Trends in Cognitive Sciences.

[11] Thioux M, Gazzola V, Keysers C (2008). Action understanding: how, what and why.. Current biology.

[12] Fogassi L, Ferrari PF, Gesierich B, Rozzi S, Chersi F (2005). Parietal lobe: from action organization to intention understanding.. Science.

[13] Ferrari PF, Gallese V, Rizzolatti G, Fogassi L (2003). Mirror neurons responding to the observation of ingestive and communicative mouth actions in the monkey ventral premotor cortex.. The European Journal of Neuroscience.

[14] Fujii N, Hihara S, Iriki A (2007). Social cognition in premotor and parietal cortex.. Social Neuroscience.

[15] Gallese V, Fadiga L, Fogassi L, Rizzolatti G (1996). Action recognition in the premotor cortex.. Brain.

[16] Keysers C, Kohler E, Umiltà MA, Nanetti L, Fogassi L (2003). Audiovisual mirror neurons and action recognition.. Experimental Brain Research.

[17] Kohler E, Keysers C, Umiltà MA, Fogassi L, Gallese V (2002). Hearing sounds, understanding actions: Action representation in mirror neurons.. Science.

[18] Rizzolatti G, Fadiga L, Gallese V, Fogassi L (1996). Premotor cortex and the recognition of motor actions.. Cognitive Brain Research.

[19] Umiltà MA, Kohler E, Gallese V, Fogassi L, Fadiga L (2001). I know what you are doing: A neurophysiological study.. Neuron.

[20] Blakemore SJ, Decety J (2001). From the perception of action to the understanding of intention.. Nature Reviews of Neuroscience.

[21] Gazzola V, Keysers C (2008). The Observation and Execution of Actions Share Motor and Somatosensory Voxels in all Tested Subjects: Single-Subject Analyses of Unsmoothed fMRI Data.. Cereb Cortex.

[22] Buccino G, Binkofski F, Fink GR, Fadiga L, Fogassi L (2001). Action observation activates premotor and parietal areas in a somatosopic manner: An fMRI study.. The European Journal of Neuroscience.

[23] Decety J, Grèzes J, Costes N, Perani D, Jeannerod M (1997). Brain activity during observation of actions. Influence of action content and subject's strategy.. Brain.

[24] Fadiga L, Fogassi L, Pavesi G, Rizzolatti G (1995). Motor facilitation during action observation: A magnetic stimulation study.. Journal of Neurophysiology.

[25] Grafton ST, Arbib MA, Fadiga L, Rizzolatti G (1996). Localization of grasp representations in humans by positron emission tomography. 2. Observation compared with imagination.. Experimental Brain Research.

[26] Iacoboni M, Woods RP, Brass M, Bekkering H, Mazziotta JC (1999). Cortical mechanisms of human imitation.. Science.

[27] Jeannerod M (2001). Neural simulation of action: A unifying mechanism for motor cognition.. Neuroimage.

[28] Rizzolatti G, Craighero L (2004). The mirror-neuron system.. Annual Review of Neuroscience.

[29] Chong TT, Cunnington R, Williams MA, Kanwisher N, Mattingley JB (2008). fMRI adaptation reveals mirror neurons in human inferior parietal cortex.. Current biology.

[30] Nelissen K, Luppino G, Vanduffel W, Rizzolatti G, Orban GA (2005). Observing others: multiple action representation in the frontal lobe.. Science.

[31] Petrides M, Cadoret G, Mackey S (2005). Orofacial somatomotor responses in the macaque monkey homologue of Broca's area.. Nature.

[32] Gazzola V, van der Worp H, Mulder T, Wicker B, Keysers C (2007). Aplasics Born without Hands Mirror the Goal of Hand Actions with Their Feet.. Current biology.

[33] Hickok G (2009). Eight problems for the mirror neuron theory of action understanding in monkeys and humans.. Journal of Cognitive Neuroscience.

[34] Poldrack RA (2006). Can cognitive processes be inferred from neuroimaging data?. Trends Cogn Sci.

[35] Buccino G, Lui F, Canessa N, Patteri I, Lagravinese G (2004). Neural circuits involved in the recognition of actions performed by nonconspecifics: an FMRI study.. Journal of Cognitive Neuroscience.

[36] Buxbaum LJ, Kyle KM, Menon R (2005). On beyond mirror neurons: internal representations subserving imitation and recognition of skilled object-related actions in humans.. Cognitive Brain Research.

[37] Gallese V, Goldman AI (1998). Mirror neurons and the simulation theory of mind-reading.. Trends in Cognitive Sciences.

[38] Gazzola V, Aziz-Zadeh L, Keysers C (2006). Empathy and the Somatotopic Auditory Mirror System in Humans.. Current biology.

[39] Gazzola V, Rizzolatti G, Wicker B, Keysers C (2007). The anthropomorphic brain: The mirror neuron system responds to human and robotic actions.. Neuroimage.

[40] Hamzei F, Rijntjes M, Dettmers C, Glauche V, Weiller C (2003). The human action recognition system and its relationship to Broca's area: an fMRI study.. Neuroimage.

[41] Heiser M, Iacoboni M, Maeda F, Marcus J, Mazziotta JC (2003). The essential role of Broca's area in imitation.. The European Journal of Neuroscience.

[42] Iacoboni M, Molnar-Szakacs I, Gallese V, Buccino G, Mazziotta JC (2005). Grasping the intentions of others with one's own mirror neuron system.. PLoS Biology.

[43] Keysers C, Gazzola V (2006). Towards a unifying neural theory of social cognition.. Progress in Brain Research.

[44] Kilner JM, Friston KJ, Frith CD (2007). Predictive coding: an account of the mirror neuron system.. Cognitive Processes.

[45] Nishitani N, Hari R (2000). Temporal dynamics of cortical representation for action.. Proceedings of the National Academy of Sciences of the United States of America.

[46] Rizzolatti G, Fadiga L, Matelli M, Bettinardi V, Paulesu E (1996). Localization of grasp representations in humans by PET: 1. Observation versus execution.. Experimental Brain Research.

[47] Ochipa C, Rothi LJ, Heilman KM (1989). Ideational apraxia: a deficit in tool selection and use.. Annals of Neurology.

[48] Mozaz M, Rothi LJ, Anderson JM, Crucian GP, Heilman KM (2002). Postural knowledge of transitive pantomimes and intransitive gestures.. Journal of the International Neuropsychological Society.

[49] Choi SH, Na DL, Kang E, Lee KE, Lee SW (2001). Functional magnetic resonance imaging during pantomiming tool-use gestures.. Experimental Brain Research.

[50] Fridman EA, Immisch I, Hanakawa T, Bohlhalter S, Waldvogel D (2006). The role of the dorsal stream for gesture production.. Neuroimage.

[51] Hermsdörfer J, Goldenberg G, Wachsmuth C, Conrad B, Ceballos-Baumann AO (2001). Cortical correlates of gesture processing: clues to the cerebral mechanisms underlying apraxia during the imitation of meaningless gestures.. Neuroimage.

[52] Higuchi S, Imamizu H, Kawato M (2007). Cerebellar activity evoked by common tool-use execution and imagery tasks: an fMRI study.. Cortex.

[53] Lotze M, Heymans U, Birbaumer N, Veit R, Erb M (2006). Differential cerebral activation during observation of expressive gestures and motor acts.. Neuropsychologia.

[54] Moll J, de Oliveira-Souza R, Passman LJ, Cunha FC, Souza-Lima F (2000). Functional MRI correlates of real and imagined tool-use pantomimes.. Neurology.

[55] Nair DG, Purcott KL, Fuchs A, Steinberg F, Kelso JA (2003). Cortical and cerebellar activity of the human brain during imagined and executed unimanual and bimanual action sequences: a functional MRI study.. Brain research Cognitive brain research.

[56] Ohgami Y, Matsuo K, Uchida N, Nakai T (2004). An fMRI study of tool-use gestures: body part as object and pantomime.. Neuroreport.

[57] Montgomery KJ, Isenberg N, Haxby JV (2007). Communicative hand gestures and object-directed hand movements activated the mirror neuron system.. Social Cognitive and Affective Neuroscience.

[58] Castelli F, Happè F, Frith U, Frith CD (2000). Movement and mind: A functional imaging study of perception and interpretation of complex intentional movement patterns.. Neuroimage.

[59] Brunet E, Sarfati Y, Hardy-Baylè MC, Decety J (2000). A PET investigation of the attribution of intentions with a nonverbal task.. Neuroimage.

[60] Ciaramidaro A, Adenzato M, Enrici I, Erk S, Pia L (2007). The intentional network: how the brain reads varieties of intentions.. Neuropsychologia.

[61] Fletcher PC, Happè F, Frith U, Baker S (1995). Other minds in the brain: A functional neuroimaging study of ‘theory of mind’ in story comprehension.. Cognition.

[62] Frith CD, Frith U (2006). The neural basis of mentalizing.. Neuron.

[63] Gallagher HL, Frith CD (2003). Functional imaging of ‘theory of mind’.. Trends in Cognitive Sciences.

[64] Gallagher HL, Happè F, Brunswick N, Fletcher PC, Frith U (2000). Reading the mind in cartoons and stories: An fMRI study of ‘theory of mind’ in verbal and nonverbal tasks.. Neuropsychologia.

[65] Gallagher HL, Jack AI, Roepstorff A, Frith CD (2002). Imaging the intentional stance in a competitive game.. Neuroimage.

[66] Hampton AN, Bossaerts P, O'Doherty JP (2008). Neural correlates of mentalizing-related computations during strategic interactions in humans.. Proceedings of the National Academy of Sciences of the United States of America.

[67] McCabe K, Houser D, Ryan L, Smith V, Trouard T (2001). A functional imaging study of cooperation in two-person reciprocal exchange.. Proceedings of the National Academy of Sciences of the United States of America.

[68] Saxe R, Kanwisher NG (2003). People thinking about thinking people. The role of the temporo-parietal junction in “theory of mind”.. Neuroimage.

[69] Sommer M, Döhnel K, Sodian B, Meinhardt J, Thoermer C (2007). Neural correlates of true and false belief reasoning.. Neuroimage.

[70] Vogeley K, Bussfeld P, Newen A, Herrmann S, Happè F (2001). Mind reading: neural mechanisms of theory of mind and self-perspective.. Neuroimage.

[71] Walter H, Adenzato M, Ciaramidaro A, Enrici I, Pia L (2004). Understanding intentions in social interaction: the role of the anterior paracingulate cortex.. Journal of Cognitive Neuroscience.

[72] Gallagher HL, Frith CD (2004). Dissociable neural pathways for the perception and recognition of expressive and instrumental gestures.. Neuropsychologia.

[73] Mitchell JP (2008). Activity in right temporo-parietal junction is not selective for theory-of-mind.. Cerebral Cortex.

[74] Decety J, Lamm C (2007). The role of the right temporoparietal junction in social interaction: how low-level computational processes contribute to meta-cognition.. The Neuroscientist.

[75] van der Gaag C, Minderaa RB, Keysers C (2007). Facial expressions: what the mirror neuron system can and cannot tell us.. Social Neuroscience.

[76] Singer T, Seymour B, O'Doherty J, Kaube H, Dolan RJ (2004). Empathy for pain involves the affective but not sensory components of pain.. Science.

[77] Oldfield RC (1971). The assessment and analysis of handedness: the Edinburgh inventory.. Neuropsychologia.

[78] Amodio DM, Frith CD (2006). Meeting of minds: the medial frontal cortex and social cognition.. Nature Reviews of Neuroscience.

[79] Brett M, Anton J, Valabregue R, Poline JB (2002). Region of interest analysis using the MarsBar toolbox for SPM 99.. Neuroimage.

[80] Brass M, Bekkering H, Wohlschläger AM, Prinz W (2000). Compatibility between observed and executed finger movements: Comparing symbolic, spatial, and imitative cues.. Brain and Cognition.

[81] Kilner J, Hamilton AF, Blakemore SJ (2007). Interference effect of observed human movement on action is due to velocity profile of biological motion.. Social Neuroscience.

[82] Iacoboni M, Lieberman MD, Knowlton BJ, Molnar-Szakacs I, Moritz M (2004). Watching social interactions produces dorsomedial prefrontal and medial parietal BOLD fMRI signal increases compared to a resting baseline.. Neuroimage.

[83] Siegal M, Varley R (2002). Neural systems involved in theory of mind.. Nature Reviews of Neuroscience.

[84] Vogeley K, Fink GR (2003). Neural correlates of the first-person-perspective.. Trends in Cognitive Sciences.

[85] Kampe KK, Frith CD, Frith U (2003). “Hey John”: signals conveying communicative intention toward the self activate brain regions associated with “mentalizing,” regardless of modality.. The Journal of Neuroscience.

[86] Raichle ME, Snyder AZ (2007). A default mode of brain function: a brief history of an evolving idea.. Neuroimage 37: 1083-1090; discussion.

[87] Pelphrey K, Morris J, McCarthy G (2004). Grasping the intentions of others: The perceived intentionality of an action influences activity in the superior temporal sulcus during social perception.. Journal of Cognitive Neuroscience.

[88] Saxe R, Wexler A (2005). Making sense of another mind: the role of the right temporo-parietal junction.. Neuropsychologia.

[89] Saxe R, Xiao D, Kovacs G, Perrett D, Kanwisher NG (2004). A region of right posterior superior temporal sulcus responds to observed intentional actions.. Neuropsychologia.

[90] Scholz J, Triantafyllou C, Whitfield-Gabrieli S, Brown EN, Saxe R (2009). Distinct regions of right temporo-parietal junction are selective for theory of mind and exogenous attention.. PLoS One.

[91] Cubelli R, Bartolo A, Nichelli P, Della Sala S (2006). List effect in apraxia assessment.. Neurosci Lett.

[92] Pazzaglia M, Pizzamiglio L, Pes E, Aglioti SM (2008). The Sounds of Actions in Apraxia.. Current biology.

[93] Rothi LJ, Heilman KM, Watson RT (1985). Pantomime comprehension and ideomotor apraxia.. J Neurol Neurosurg Psychiatr.

[94] Thompson JC, Hardee JE, Panayiotou A, Crewther D, Puce A (2007). Common and distinct brain activation to viewing dynamic sequences of face and hand movements.. Neuroimage.

[95] Wheaton KJ, Thompson JC, Syngeniotis A, Abbott DF, Puce A (2004). Viewing the motion of human body parts activates different regions of premotor, temporal, and parietal cortex.. Neuroimage.

