# Supplementary material for: Playing Charades in the fMRI: Are Mirror and/or Mentalizing Areas Involved in Gestural Communication?
Source: PLoS One. 2009 Aug 27;4(8):e6801. doi: 10.1371/journal.pone.0006801 (PMC2728843; doi:10.1371/journal.pone.0006801)
Supplement: Text S1 — Does the MNS need objects to be activated? Some studies have investigated whether the MNS can respond to actions not directed at objects. In this supporting information we discuss the question whether the current study can provide further insights into this question. (0.04 MB DOC) [file pone.0006801.s001.doc]

## Supporting Information 1

## Does the MNS need objects to be activated?

Some studies have investigated whether the MNS can respond to actions not directed at objects [e.g. 1,2]. Can the current study provide further insights into this question? While it is true that none of our stimuli had an object physically present, all of the words people had to mime referred to objects (Tab. 1 in main article). Accordingly, the production and observation of our gestures may have involved mentally filling in objects that were implied by the gestures. Single-cell recordings in the monkey [2] support this view: implying the presence of objects can make mirror neurons selective to viewing actions for which the object is not physically present. In monkeys, implying the presence of an object cannot be done by miming the action without the object (which did not trigger activity in Umiltà et al., 2001 [2]), but can be achieved by placing an occluding screen in front of the object. Humans, unlike monkeys, routinely engage in “let’s pretend” play as children [3]. This raises the question of whether miming might be more effective at implying the presence of objects to the MNS. Buccino et al., [1] showed that most of the pMNS seems to respond to the sight of grasping a cup but not when the cup was absent. Our data however shows activity in those parietal nodes of the pMNS in which Buccino et al., [1], found activity only for the movies including the object. This may suggest that a simple mimed grasp may fail to conjure up a mental object, but that within the context of our experiment, more elaborate gestures simulating the presence of an object may be more effective. In conclusion, the apparently simple question of whether the pMNS responds to actions not directed to physically present objects becomes more complex if one considers that mimed actions may differ in their effectiveness at conjuring mental representations of objects. A similar difficulty applies to communicative gestures (e.g. Montgomery, Isenberg, & Haxby, 2007, [4], “come here!” gesture), which always imply another person as a target. However, what our experiment does show is that in the context of deliberate gestural communication, gestures can recruit the pMNS even if their object is not physically present.

1. Buccino G, Binkofski F, Fink GR, Fadiga L, Fogassi L, et al. (2001) Action observation activates premotor and parietal areas in a somatosopic manner: An fMRI study. The European Journal of Neuroscience 13: 400-404.

2. Umiltà MA, Kohler E, Gallese V, Fogassi L, Fadiga L, et al. (2001) I know what you are doing: A neurophysiological study. Neuron 31: 155-165.

3. Fein GG (1981) Pretend Play in Childhood: An Integrative Review. Child Development.

4. Montgomery KJ, N.Isenberg, Haxby JV (2007) Communicative hand gestures and object-directed hand movements activated the mirror neuron system. Social Cognitive and Affective Neuroscience 2: 114-122.
