# Supplementary figures and images for: Playing Charades in the fMRI: Are Mirror and/or Mentalizing Areas Involved in Gestural Communication?
Source: PLoS One. 2009 Aug 27;4(8):e6801. doi: 10.1371/journal.pone.0006801 (PMC2728843; doi:10.1371/journal.pone.0006801)

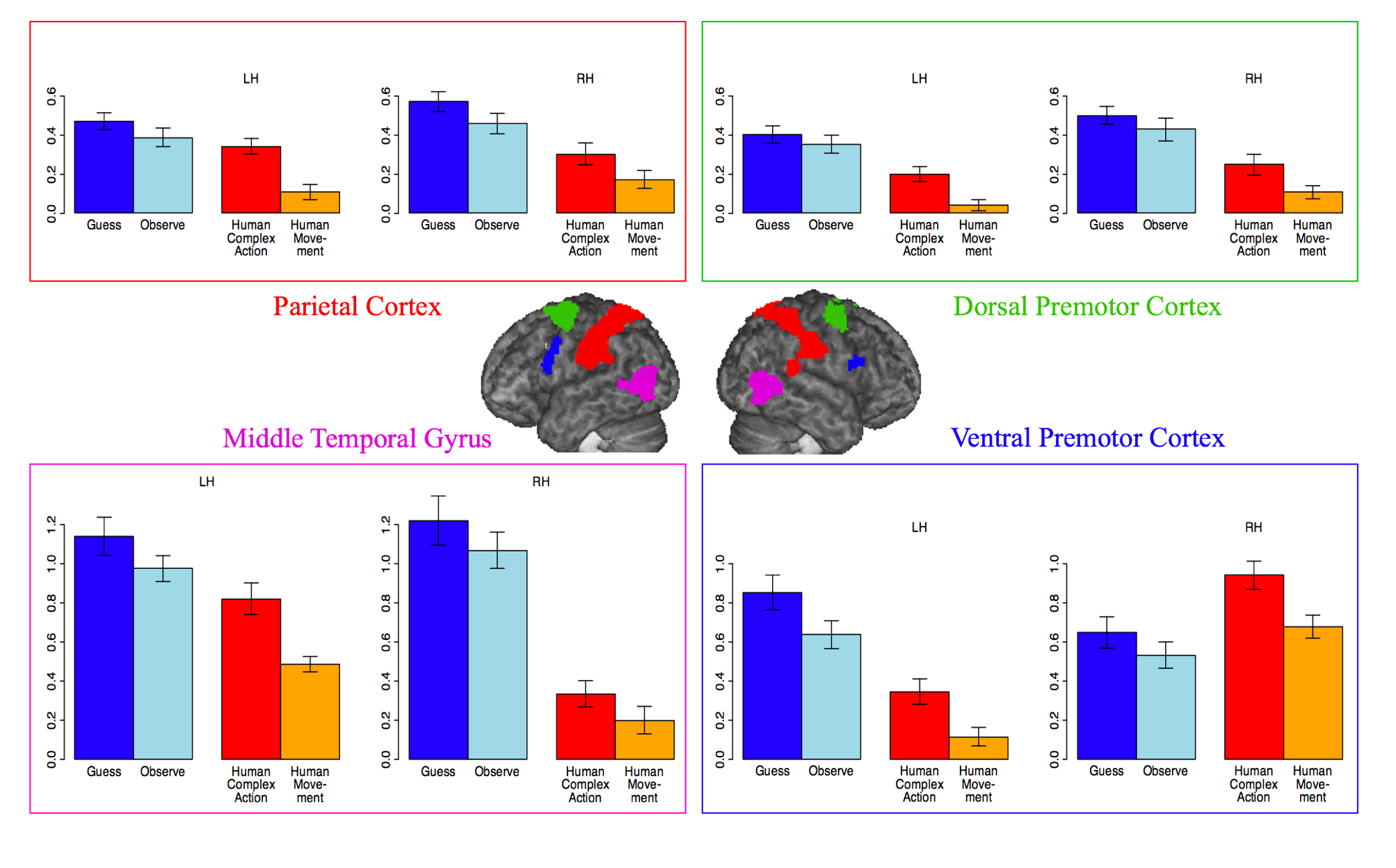

Supplement: Figure S1 — Comparison with Gazzola et al., 2007. Comparison of mean percent signal change during gesture observation (light and dark blue bars) with those during the observation of goal directed actions (red and orange, Gazzola et al., 2007). The blocks of action observation differed across experiments: over 50 s in the current experiment and 13.5 seconds in Gazzola et al., 2007. Instead of comparing parameter estimates over the entire period of observation, we therefore extracted the mean percent signal change at the moment (16 s) in which activity to the shorter of the two blocks (Gazzola et al., 2007) peaked. The bar graphs represent the mean percent signal change at 16 s post stimulus onset (±s.e.m.) separately for Guessing (dark blue) and Passive Observation (light blue) from the current study and for the observation of a hand manipulating an object (red) and a hand moving to rest on a table without manipulating an object (orange) from the data of Gazzola et al., 2007. ROIs are shown in the centre. (7.51 MB TIF) [file pone.0006801.s002.tif]
